# The Stepwise Behavioral Responses: Behavioral Adjustment of the Chinese Rare Minnow (*Gobiocypris rarus*) in the Exposure of Carbamate Pesticides

**DOI:** 10.1155/2013/697279

**Published:** 2013-07-16

**Authors:** Zongming Ren, Liang Liu, Rongshu Fu, Mingsheng Miao

**Affiliations:** College of Life Science, Shandong Normal University, Ji'nan 250014, China

## Abstract

In order to illustrate the behavioral regulation in environmental stress, the behavioral responses of the Chinese rare minnow (*Gobiocypris rarus*) to arprocarb, carbofuran, and oxamyl were analyzed with an online monitoring system. The Self-Organizing Map (SOM) was used to define the patterns of the behavioral data obtained from treatments at concentrations of 0.1 toxic unit (TU), 1 TU, 2 TU, 5 TU, 10 TU, and 20 TU and a control. In certain cases, differences among the carbamate pesticides (CPs) tested were observed. The profiles of behavioral strength (BS) in SOM varied according to the concentration used. The time of the first significant decrease of the BS varied inversely with the CP concentrations. The results suggested that the behavioral regulation in the stepwise behavioral responses (SBR) was evident. The primary movement behaviors shown by the SBR model included no effect, stimulation, acclimation, adjustment (readjustment), and toxic effect, especially at the lower concentrations. However, higher stress (10 TU and 20 TU) might limit the function of the behavioral adjustment produced by the intrinsic response mechanisms. It was concluded that SBR, which were affected by both the concentration and the exposure time, could be used as a suitable indicator in the ecotoxicological risk assessment of CPs.

## 1. Introduction

Carbamate pesticides (CPs) developed and applied as insecticides have long been in use. Their widespread application has caused varying amounts of pollution in many rivers and lakes. These compounds can inhibit cholinesterase (ChE) [1] and produce unregulated nerve ending activation and paralysis in organisms [2]. These pesticides may then impact populations and biological communities [3, 4]. CPs can even cause severe poisoning in humans. These observations suggest that CP pollution of the environment is a serious problem that requires increased attention [5–7]. Therefore, it is necessary to gain a clear understanding of the potential threats to human health and the ecological balance of aquatic ecosystems posed by the presence of these substances in the aquatic environment.

The behavioral effects of CPs suggest that behavioral responses can be used as a suitable indicator in the assessment of the toxicological impacts of CPs at the entire test endpoints used for such assessment [8–10]. These pesticides may cause hyperactivity, loss of coordination, convulsions, paralysis, and other types of behavioral changes due to ChE inhibition [11]. According to previous research [12–17], behavioral responses to contaminants serve an adaptive function by reducing exposure to harmful conditions, whereas failure to avoid exposure may result in reduced fitness and survival and eventually produce detrimental effects. The intrinsic adjustment of the internal environment at different levels might induce visible stepwise behavioral responses [18], which included behavior stimulation, behavior acclimation, and behavior adjustment [19].

The Chinese rare minnow (*Gobiocypris rarus*) is used as a model animal in toxicological studies and belongs to the large family Cyprinidae. These fish are primarily found in the upstream reaches of the Yangtze River, Sichuan Province, China. The fish are small (30–80 mm in total length) and easy to culture in the laboratory. Their relatively short life cycles, their production of hundreds of eggs with high fertilization rates, and their hatching rates make them the perfect model fish for the laboratory [20, 21]. Many studies related to rare minnow toxicity have appeared [22, 23]. However, few reports have addressed the behavioral toxicology of the rare minnow. A possible reason for this lack of study is that the progress of behavioral toxicology as a consensus-based discipline has been hindered by a lack of test standards, by the homogeneity of samples and by the variation in measured endpoints [24, 25].

It is probable that the extensive use and discharge of CPs in the environment will continue in the foreseeable future. In order to illustrate the behavioral regulation of the rare minnow in CP stress, the current study was therefore performed to assess the effects of CPs on the aquatic environment by examining the behavioral effects of arprocarb, carbofuran, and oxamyl with an online monitoring system and Self-Organizing Map analysis. The online monitoring system was built at the Chinese Academy of Sciences [26], and Self-Organizing Map performs a nonlinear projection of data onto a two-dimensional space and provides a patterned map of the input data [27]. This analysis could evaluate the behavioral responses of the test species based on behavior strength (BS). Furthermore, the stepwise behavioral response model of the rare minnow under CP stress was discussed. Experiments with continuous treatments and computational methods were used to objectively characterize complex behavioral data in response to different chemicals and concentrations.

## 2. Materials and Methods

### 2.1. Equipment

**Figure 1:**
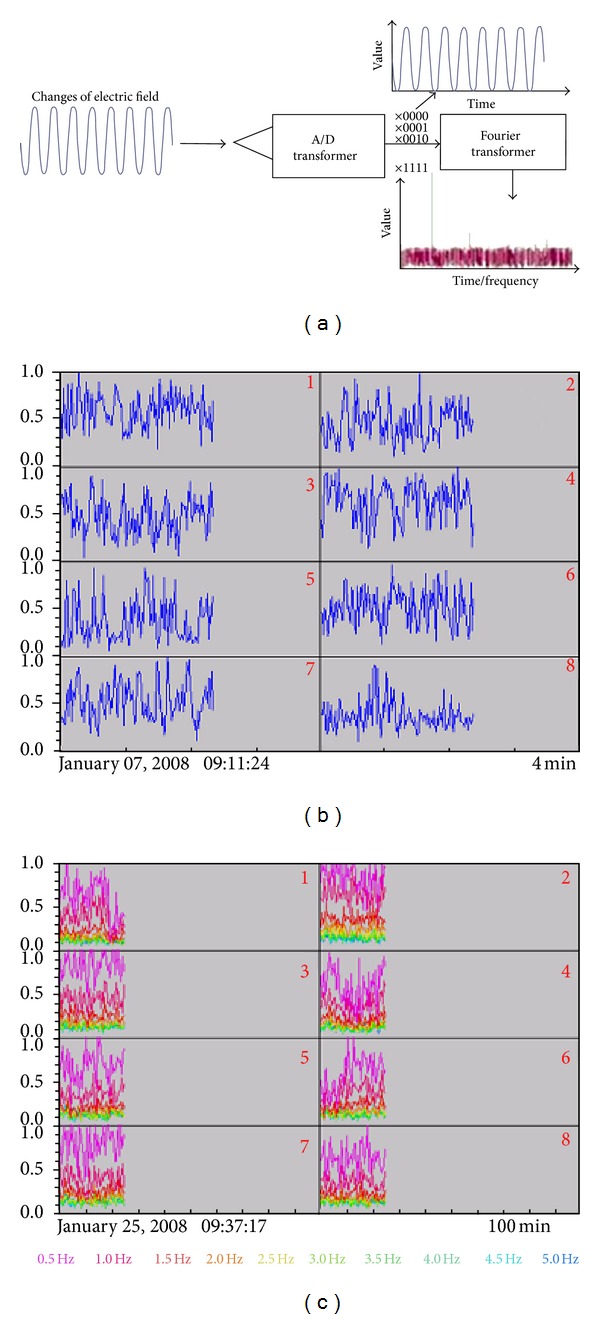
Signal acquisition and transmission in the online monitoring system. (a) Signal acquisition in the online monitoring system; (b) normal signal analysis (BS); and (c) signal analysis after fast Fourier transform processing.

The behavioral response of the rare minnow was monitored with an online monitoring system built at the Chinese Academy of Sciences [26]. The test organisms were placed in a flow-through test chamber (7 cm long, 5 cm in diameter) closed at both sides with nylon nets (250 *μ*m). One pair of electrodes on the walls of the test chamber transmits a high-frequency alternating-current signal. This signal is received by a second pair of noncurrent-carrying electrodes [28]. The BS of the test organism is converted to digital form by an A/D converter. The changes in the output signal from the A/D converter were analyzed automatically by software attached to the equipment (Figure 1). The BS is sampled automatically every second by the online monitoring system, and the average BS value calculated every 6 minutes is used to analyze the behavioral changes by comparing the sample values with those in the online monitoring system control database. BS values varying from 0 (loss of the ability to move) to 1 (full behavior expressed) were used to indicate the differences in the behavioral responses of the rare minnow. The standard used to determine a significant decrease in BS (SD-BS) was that the approximate difference in the mean values of the BS (averaged over 30 min of observations) represented a change of no less than 20% [29].

### 2.2. Test Species

The rare minnows used in this study were graciously provided by the Institute of  Hydrobiology, Chinese Academy of Sciences (Wuhan, China). The brood stock was raised in a flow-through system with dechlorinated tap water (using active carbon) and has been maintained in our laboratory for more than 4 years. During breeding, the fish were subjected to a photoperiod of 16 h light and 8 h dark at 25 ± 1°C. The brood stock was fed newly hatched brine shrimp (*Artemia*) in the morning and granule food (Trea, Germany) in the afternoon.

### 2.3. Test Chemicals

Arprocarb, carbofuran, and oxamyl were purchased from J&K Chemical Ltd. All compounds were technical grade (>95% purity). Stock solutions (stored at 4°C until use) with a proper concentration of each chemical were prepared in dimethyl sulfoxide (DMSO). Appropriate aliquots were used to prepare test solutions of specific concentrations. All solvents were of analytical grade. The concentration of  DMSO was less than 0.5% in all experiments, a concentration that neither produces acute toxicity to the rare minnow nor affects the mobility of the experimental animals [30].

### 2.4. Experimental Procedures

Behavioral response monitoring was performed under flow-through conditions. Eight test chambers were selected in this study. According to previous studies [25, 31], three healthy rare minnows (2 months old) approximately 2.5–3.0 cm in length were selected at random for each test chamber. The flow rate through each test chamber was controlled at approximately 2 liters per hour, a value previously shown to have no effect on the motility of test organisms [32]. No food was added during these experiments. A controlled photoperiod of 16 h light (04:00–20:00) and 8 h dark (20:00–04:00) was used in the experiments. 

A 48-hour experiment in which the test animals were exposed to arprocarb, carbofuran, or oxamyl was used to investigate the stepwise behavioral responses of the rare minnow. Based on the acute toxicity of these three CPs in the rare minnow, the 48-hour median lethal concentrations (LC_50_-48) were 7.5 mg/L, 0.75 mg/L, and 1.7 mg/L for arprocarb, carbofuran, and oxamyl, respectively. The chemical toxic unit (TU) for the test organisms was used for comparison; that is, the LC_50_-48 was taken as one unit (1 TU). A value of  10% LC_50_ was considered a sublethal concentration [33]. Accordingly, the 48-hour exposure tests under flow-through conditions with online chemical-mix equipment were performed for 6 concentrations (0.1 TU, 1 TU, 2 TU, 5 TU, 10 TU, and 20 TU) and repeated three times for each 48-hour exposure to compare with the control. The calculation of the TU value was as follows [33]:

(1) $$\begin{array}{c} \text{TU}=\sum \frac{C_{i}}{\text{L}{\text{C}}_{50}^{i}\text{-}48}, \end{array}$$

where *C*_*i*_ was the total concentration of chemicals and LC_50_^*i*^-48 was the 48-hour median lethal concentration.

### 2.5. Data Analysis

Initially, the behavior data was analyzed by a 3D Surface Plot of MATLAB 2009 (1984–2009 The MathWorks, Inc.). Surf (*X*, *Y*, *Z*) creates a shaded surface using *Z* for the data as well as surface height. *X* and *Y* are vectors defining the *x* and *y* components of a surface. Length (*X*) = *n* and length (*Y*) = *m*, where [*m*, *n*] = size (*Z*). In this case, the vertices of the surface faces are (*X*(*j*), *Y*(*i*), *Z*(*i*, *j*)) triples.

Self-Organizing Map (SOM) was subsequently used to classify movement patterns by training the continuous movement data of electric signal.

A Self-Organizing Map performs a nonlinear projection of data onto a two-dimensional space and provides a patterned map of the input data. This map is produced by training with unsupervised learning [27]. The size of the Self-Organizing Map was determined heuristically in a way that would be comprehensible to the reader in a smaller number of dimensions. The highest variance in the input data will be projected along the vertical axis, whereas the next highest variance would be represented on the horizontal axis. The optimal size of the computational modes was adjusted based on the degree of discrimination among the grouped nodes after training. Approximately two-thirds of the total nodes were allocated to the samples, whereas one-third of the nodes were empty and served as delimiters between the occupied nodes. Based on the results of preliminary training, a 16 × 16-node configuration was used in this study.

The Euclidean distance (*d*_*j*_(*t*)) between the weight at iteration time *t* and the input vector at the *j*th node on the Self-Organizing Map was calculated with a learning process:

(2) $$\begin{array}{c} d_{j}\left(t\right)=\sum_{i=0}^{P-1}{[x_{i}-w_{ij}\left(t\right)]}^{2}, \end{array}$$

where *x*_*i*_ is the value of the *i*th parameter, *w*_*ij*_(*t*) is the weight between the *i*th parameter and the *j*th node on the Self-Organizing Map, and *P* is the number of the parameter.

The best-matching neuron, corresponding to the minimum distance, was selected as the winner of the competition between neurons at the current iteration. For the best-matching neuron and its neighborhood neurons, the new weight vectors are updated as follows:

(3) $$\begin{array}{c} w_{ij}\left(t+1\right)=w_{ij}\left(t\right)+\alpha \left(t\right)\left[x\left(t\right)-w_{ij}\left(t\right)\right], \end{array}$$

where *t* is the iteration time and *α*(*t*) is the learning rate. The learning process for the Self-Organizing Map was computed with the Self-Organizing Map Toolbox developed by the Laboratory of Information and Computer Science, Helsinki University of Technology, in MATLAB environments [34]. The initialization and training processes followed the suggestions furnished by the Self-Organizing Map Toolbox by allowing optimization in the algorithm. A detailed description of the application of the Self-Organizing Map technique to behavioral data can be found in related reports [35].

Because the input data were provided to the Self-Organizing Map for training (3), the weights of the best-matching unit and the neighboring computational nodes were adjusted towards the input vector with interactive calculation. To reveal the degree of association between the Self-Organizing Map units, the Ward linkage method was used to cluster the movement data based on the dendrogram according to the Euclidean distance [36, 37]. The linkage distances were rescaled to a range of 0%–100%.

Based on the solutions presented by Rabiner [38], the process was applied with the programs provided in the HMM toolbox (MATLAB7.8, The Math Works, R2009).

## 3. Results and Discussion

### 3.1. The Behavioral Responses of the Rare Minnow

**Figure 2:**
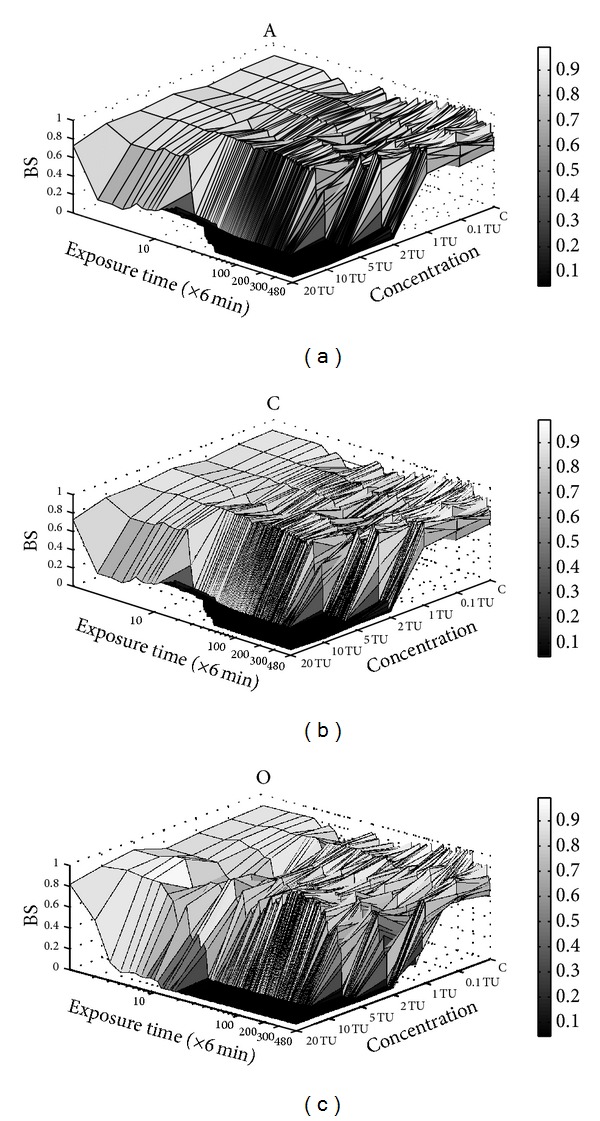
The effects of different treatments on the behavioral responses of the rare minnow at different times. Exposure time is shown on a logarithmic scale. The BS values were used in a statistical analysis of the tendencies shown by the behavioral responses of the rare minnow to different CPs. A, C, and O indicate the individual effects of arprocarb, carbofuran, and oxamyl on the behavioral responses of the rare minnow.

The effects of arprocarb, carbofuran, and oxamyl on the behavioral responses of  the rare minnow at different exposure times were shown in Figure 2. The value of  the BS in the control remained at approximately 0.8, a value similar to that occurring at the beginning of every experimental exposure to a pesticide (i.e., the first 6 min). These results showed that almost no negative effects on the behavioral responses occurred in the control or during the first 6 min of pesticide exposure. In the treatments with the same pesticide, the concentration and the exposure time evidently affected the behavioral responses, which showed a stepwise change. This finding indicated that a higher concentration and a longer exposure time could produce a relatively marked decrease in the behavioral responses. The same tendency was observed in every treatment. This result suggested that different concentrations and different exposure times produced different values of the BS. The stepwise behavioral responses were more evident at the lower concentrations (0.1 TU, 1 TU, and 2 TU) than at the higher concentrations (10 TU and 20 TU). A possible explanation for these findings is that at the higher concentrations, the intrinsic responses were not strong enough to adjust the internal environment behaviorally to adapt to the external stress [18].

The results of the study suggested that the behavioral responses of the rare minnow to different CPs tended to be similar. In all of the experimental treatments, the BS decreased to less than 0.1. This decrease was followed by a loss of movement. The behavioral responses in these situations did not show a steady decrease. Instead, several adjustments or readjustments occurred. These observations indicated that stepwise modulation represented the behavioral response to the experimental pesticide treatment.

These results suggested that the different environmental stresses occurring during different exposure periods determined the behavioral responses of the rare minnow. At the lower concentrations, for example, 0.1 TU and 1 TU, the stepwise behavioral response was more significant. This response consisted primarily of stimulation, acclimation, and adjustment (readjustment) and did not include toxic effect. The 5 TU treatments produced a substantial number of changes in the behavioral responses. These altered behavioral responses primarily included stimulation, acclimation, adjustment (readjustment), and toxic effect [39]. At the highest concentrations (10 TU and 20 TU), the rare minnow could adapt only with difficulty.

The findings of the study showed that the stepwise behavioral responses of the rare minnow were affected by both the exposure time and the pesticide concentrations. Furthermore, these findings showed that higher environmental stress might limit the ability of the behavioral adjustments generated by intrinsic response mechanisms to produce a functional outcome.

**Figure 3:**
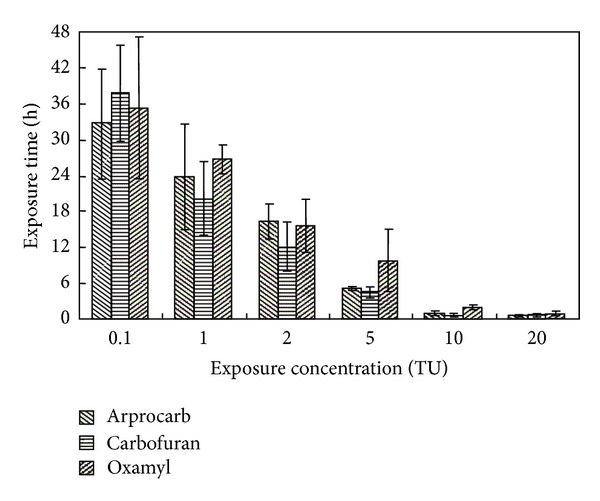
The elapsed time until the first SD-BS in the rare minnow at different concentrations of CPs. *Shown in mean ± S.D., *n* = 3.

The elapsed times from the initiation of the pesticide exposure to the occurrence of the first significant decrease in BS (SD-BS) are shown in Figure 3. Although the differences among individual SD-BS values were large relative to the standard deviations, the overall tendency was the same. These results suggested that the time until the first SD-BS values depended strongly on the pesticide concentrations. The first SD-BS was similar for the same concentrations of different CPs.

After the first SD-BS, the behavioral responses of the rare minnow differed at different pesticide concentrations. At 10 TU and 20 TU, the BS decreased abruptly to less than 0.1. This decrease suggested a possible loss of movement. However, significant stepwise modulation appeared at 5 TU, 2 TU, 1 TU, and 0.1 TU. The movements associated with the modulation included acclimation, adjustment, and readjustment. If the movement-based regulation could not satisfy the intrinsic requirements for an effective response to environmental stress (2 TU and 5 TU), significant toxic effects would occur (Figure 2). These results showed that stepwise behavioral responses were very important for the adaptation of the rare minnow to environmental stress, especially at low pesticide concentrations.

### 3.2. The Patterns of the Behavioral Responses of the Rare Minnow

**Figure 4:**
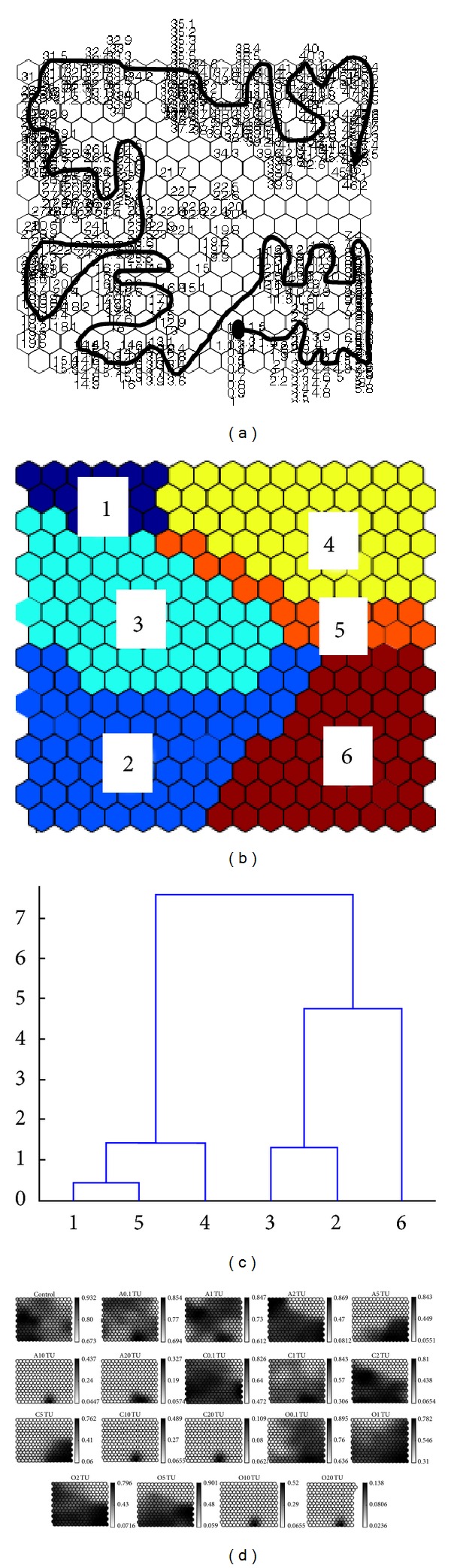
Clustering of BS on the Self-Organizing Map for different CPs and different concentrations. (a) Clusters with time series (black dot indicates the starting position); (b) six clusters classified by the Self-Organizing Map; (c) cluster distances according to the Ward linkage method; (d) profiles of BS values visualized on the Self-Organizing Map in different CPs. A 0.1 TU means exposure to 0.1 TU arprocarb. The values shown in the vertical bar indicate the range of the mean values of the BS.

The results of the Self-Organizing Map analysis showed the patterns of behavioral response of the rare minnow to the CPs and to the control (Figure 4). The exposure time was consistent with the photoperiod (Figure 4(a)). The cluster analysis identified six groups based on the mean values of the BS (Figures 4(b) and 4(c)). Cluster 6, at the bottom right corner of the Self-Organizing Map, represented the initial treatment period. Clusters 2, 3, 1, and 4 represented subsequent exposure times. Cluster 5 contained empty nodes, which served as delimiters between the occupied nodes of clusters 1, 2, 3, 4, and 6. The cluster distances calculated with the Ward linkage method indirectly suggested closeness between the groups identified by clustering (Figure 4(c)).

The profiles of the BS values visualized with the Self-Organizing Map for the CPs tested are shown in Figure 4(d). The values shown on the vertical bars indicate the ranges of the mean BS values for different concentrations and time periods. In the control group, the BS values ranged between 0.673 and 0.932. Low BS values were only observed at the end of the experiment. These values might have resulted because no food was provided over the 48-hour experimental period. Although the BS values differed somewhat among different CPs, a BS gradient was observed for different concentrations. The profiles of the BS on the Self-Organizing Map varied with the pesticide concentration. At low pesticide levels, different pesticides showed different profiles. However, the values of BS were also lower than in control, and even sublethal concentrations could induce toxic behavioral effects. Significant decreases in the BS occurred in cluster 3 at the 2 TU exposure, in clusters 2 and 6 at the 5 TU exposure, and in cluster 6 at the 10 TU and 20 TU exposures. The BS value decreased to less than 0.1 soon after the beginning of the 10 TU and 20 TU exposures.

### 3.3. The Model of the Stepwise Behavioral Responses of the Rare Minnow

**Figure 5:**
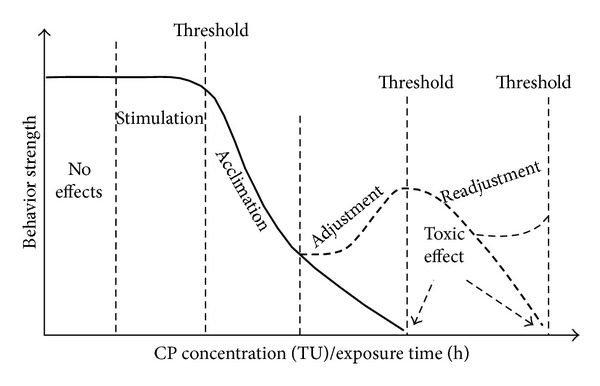
Stepwise behavioral response model for the rare minnow exposed to CPs.

Derived from the results of this study, the observations of the stepwise behavioral responses of the rare minnow suggested that the successive behavioral movements observed in different treatments could be represented by the following steps: no effect, stimulation, acclimation, adjustment (readjustment), and/or toxic effect (Figure 5). These results were consistent with those of previous studies on *Daphnia magna* [25, 28, 39], showing that increases in either the toxicant concentration or the exposure time produced a cascade of regulatory behavioral stress responses that were activated and performed by the organisms. The stepwise behavioral responses of the rare minnow can be interpreted according to the hypothesis that the organisms displayed a time-dependent sequence of different regulatory or compensatory behavioral stress responses during exposure to a pollutant at levels that exceeded the organism's threshold of resistance. The stimuli associated with increasing stress elicited regulatory responses (loading stress). Above a certain stimulus level, however, several reactions could potentially occur: (1) homeostasis could not be maintained, and the organism suffered toxic effects (limiting stress); (2) the organism could acclimate to the increased stress level, or (3) the organism decreased the performance of the response and increased the performance of another response to the stimulus. If the first stress response decreased to less than the original level, a toxic effect occurs.

In theory, the first behavioral modulation shown by the rare minnow comprised an increase in the strength of all movements, presumably to attempt to escape from the polluted aquatic environment (the avoidance behavior) [28]. This concept explains the behavioral responses observed in stimulation, the step immediately following no effect. Presumably, the stress caused by certain concentrations of carbamate pesticides would be too high to allow the rare minnow to begin the avoidance behavior. This type of behavior was absent or of short duration at high CP concentrations (Figure 2). In these cases, the BS tended to decrease gradually until the ability for movement was lost. In other cases, the rare minnow could apparently cope with the neurotoxin stressors and increased its BS. Associated with this behavioral response, the length of stimulation was close to the expected value, approximately 1 to 3 hours (Figure 3), and varied inversely with the pesticide concentrations.

Stimulation was usually followed by acclimation. The BS became steadily weaker during acclimation. The primary reason for the decrease in movement behavior during acclimation was that the behavioral adjustment reached an extreme level (“alarm reaction”) (Threshold 1). Over time, stress gradually decreased the motility of the test organisms.

After acclimation, two behavioral responses were possible. If the rare minnow could not overcome threshold 1, toxic effect would occur. Alternatively, if the rare minnow survived through threshold 1, the BS returned in a short time, as shown in adjustment. A second extreme, “alarm reaction” (Threshold 2) would occur preceding toxic effect.

Based on these results, “avoidance behavior” was an appropriate concept to characterize the stepwise behavioral responses, as reported in previous research [28]. Almost all aquatic animals can actively escape from a polluted environment and move to an unpolluted area. The stepwise behavioral responses of the rare minnow also showed a tendency for behavioral modulation to maintain a stable internal environment and diminish the dependence on the external environment.

According to previous research of Stepwise Stress Model (SSM), stepwise behavioral responses were found in both *D. magna *and *G. holbrooki* when exposed to different chemicals. *D. magna* decreased locomotion and ventilation (first step), followed by increased ventilation (second step). *G. holbrooki *decreased locomotion (first step) and increased ventilation at intermediate pH levels (second step) [19, 39]. Meanwhile, increasing environmental stress will result in more intensive behavioral responses of *D. magna* and shorter response time [39]. The model of the stepwise behavioral responses of the rare minnow during the pesticide exposure supported and developed SSM, which included no effects, stimulation, acclimation, adjustment (readjustment), and toxic effect. In this study, the stepwise behavioral responses model showed the effects of both the CP concentrations and the exposure time. In the SSM, however, the stepwise behavioral responses model only showed changes in the BS at one concentration as a function of the exposure time, which was reflected by a time series BS data in one treatment. In contrast, the presence of thresholds mediated the effects of environmental stress on the behavioral responses of the rare minnow and determined the tendencies shown by the behavioral responses.

## 4. Conclusion

The toxicity characteristics of all CPs on the movement behavior of organisms were the same because these pesticides are ChE inhibitors [1]. The restraint activity via ChE inhibition resulted in unregulated nerve ending activation and paralysis in organisms [2]. These effects could induce a loss of nerve conduction ability and subsequently cause hyperactivity, a loss of coordination, convulsions, paralysis, and other types of behavioral changes. All of these behavioral disorders could produce stepwise behavioral responses. Therefore, the stepwise behavioral responses, which could be suitable for use as an indicator in the ecotoxicological risk assessment of CPs, would be affected by both the pesticide concentrations and the exposure time (Figure 5). The movement-strength data (BS) mining with the Self-Organizing Map technique and the stepwise behavioral response processes could be used efficiently in combination to illustrate the characteristics of the behavioral processes that occur and to monitor toxic chemicals in the environment.

Because the restraint on activity produced by ChE may have caused the stepwise behavioral responses of the rare minnow [40], further investigations should focus on the relationship between the inhibition of ChE and the behavioral responses through the use of in vivo testing to examine the intrinsic response mechanism characterizing the stepwise model.
